# Influence of Natural Polysaccharides on Properties of the Biomicroconcrete-Type Bioceramics

**DOI:** 10.3390/ma14247496

**Published:** 2021-12-07

**Authors:** Piotr Pańtak, Ewelina Cichoń, Joanna Czechowska, Aneta Zima

**Affiliations:** Faculty of Materials Science and Ceramics, AGH University of Science and Technology, Mickiewicza Av. 30, 30-058 Kraków, Poland; pantak@agh.edu.pl (P.P.); ecichon@agh.edu.pl (E.C.); jczech@agh.edu.pl (J.C.)

**Keywords:** biomicroconcretes, hybrid materials, calcium phosphate, chitosan, dual setting

## Abstract

In this paper, novel hybrid biomicroconcrete-type composites were developed and investigated. The solid phase of materials consisted of a highly reactive α -tricalcium phosphate (α-TCP) powder, hybrid hydroxyapatite-chitosan (HAp-CTS) material in the form of powder and granules (as aggregates), and the polysaccharides sodium alginate (SA) or hydroxypropyl methylcellulose (HPMC). The liquid/gel phase in the studied materials constituted a citrus pectin gel. The influence of SA or HPMC on the setting reaction, microstructure, mechanical as well as biological properties of biomicroconcretes was investigated. Studies revealed that manufactured cement pastes were characterized by high plasticity and cohesion. The dual setting system of developed biomicroconcretes, achieved through α-TCP setting reaction and polymer crosslinking, resulted in a higher compressive strength. Material with the highest content of sodium alginate possessed the highest mechanical strength (~17 MPa), whereas the addition of hydroxypropyl methylcellulose led to a subtle compressive strength decrease. The obtained biomicroconcretes were chemically stable and characterized by a high bioactive potential. The novel biomaterials with favorable physicochemical and biological properties can be prosperous materials for filling bone tissue defects of any shape and size.

## 1. Introduction

The bone substitutes constitute an important group of biomaterials widely used in orthopedics, craniofacial surgery, and dentistry [1]. Due to the growing demand for implantable materials, researchers are constantly working on improving their properties as well as on developing completely new bone substitutes [2]. Calcium phosphate bone cements (CPCs) are biocompatible, moldable materials, which harden in vivo through a low temperature setting reaction. CPCs, in addition to biocompatibility, bioactivity, and mechanical properties similar to bone tissue, should also possess appropriate rheological properties, injectability, cohesion, and setting times. A major advantage of CPCs over the preformed grafts is their ability to adapt to the complex geometry of the bone defect. Despite the excellent biocompatibility and bioactivity of CPCs, their poor mechanical strength and frequent lack of satisfactory injectability limit their applications [3,4]. In order to overcome poor injectability and compressive strength of CPCs, various polymeric additives, including polyanions (e.g., pectins, sodium alginate, and methylcellulose) and polycations (e.g., chitosan and CTS) can be applied. From the point of view of bone tissue engineering, sodium alginate (SA) and hydroxypropyl methylcellulose (HPMC) have favorable properties (i.e., biocompatibility, low toxicity, and ease of gelation). SA belongs to the group of alginates derived from brown algae cell walls. This anionic polysaccharide is widely used in pharmaceutical [5] and biomedical applications [6,7,8]. In the field of calcium phosphate-based bone cements, SA is often applied because materials with sodium alginate gain injectability and show beneficial properties in in vitro tests [9,10]. HPMC (anionic polysaccharide) is a cellulose-derived polymer with a wide range of biomedical applications [11]. In the studies of Burguera et al. [12], the HPMC to the liquid phase of cements allowed to obtain CPCs with better injectability, and Liu et al. [13] pointed out the outstanding cohesion of bone cements containing HPMC. An additional reason for the use of the above-mentioned polymers is their biologically favorable features, such as biocompatibility, biodegradability, and antimicrobial properties [14,15,16,17].

An interesting modification of bone cements seems to be biomicroconcretes, i.e., bone cements containing aggregates in the form of microspheres or granules [18,19,20,21,22,23]. Nezafati et al. [23] investigated tetra calcium phosphate (TTCP) based cement with gelatine microspheres (GMs). They stated that the final setting time and injectability were increased when GMs were added to the CPCs. Meng et al. [21] examined material composed of α-tricalcium phosphate (α-TCP), calcium dihydrogen phosphate monohydrate (DCPM), and CaCO_3_ incorporated with chitosan microspheres (10%, *w*/*w*). They demonstrated that the biomicroconcretes promote adhesion, proliferation, and differentiation of osteoblasts and had good biocompatibility in the muscles of animals. In the study of Zima et al., biomicroconcretes based on α-TCP and hybrid, hydroxyapatite-chitosan (HAp-CTS) granules were bioactive and supported the growth of bone tissue [18]. Furthermore, the use of chitosan (polycation) together with polyanionic polymers is of great interest due to their unique electrostatic interaction in an aqueous environment [24,25,26]. Neufeld et al. [27] studied pectin/chitosan physical hydrogels as potential drug delivery vehicles. Dziadek et al. [28] combined α-TCP, hybrid HAp/CTS granules, and pectins receiving injectable biomicroconcretes with improved surgical handiness, due to polyelectrolyte complex formation. The use of pectins in cement-based bone substitutes is still under evaluation. The crosslinking agents have also been studied for the fabrication of particles or composites from chitosan and pectin [29,30]. In this study, polymeric additives, i.e., sodium alginate (SA) and hydroxypropyl methylcellulose (HPMC) as a polyanions will be examined as potential moderators of interactions between polycationic chitosan and citrus pectin. We suspect that the use of polyanionic polymers together with polycationic chitosan can lead to achieving favorable properties of hybrid, biomicroconcrete-type materials due to the formation of polyelectrolyte complexes.

The aim of this study was to develop and obtain biomicroconcretes on the basis of α-tricalcium phosphate, hydroxyapatite, chitosan, and citrus pectin and investigate the influence of natural polysaccharides in the form of sodium alginate and hydroxypropyl methylcellulose (2 and 4 wt%) on their physicochemical and biological properties.

## 2. Materials and Methods

### 2.1. Materials

The initial α-tricalcium phosphate (α-TCP) powder was synthesized by the wet chemical method described previously [31,32]. As reagents, Ca(OH)_2_ (≥99.5%, POCH, Gliwice, Poland) and H_3_PO_4_ (85.0%, POCH, Gliwice, Poland) were applied. In brief, the α-TCP precipitate was dried, sintered above 1250 °C (5 h), ground in an attritor mill (3 h), and sieved below 63 µm.

Hydroxyapatite-chitosan (HAp-CTS) hybrid materials, containing 21 wt% of chitosan, were obtained by the modified wet chemical method described previously [33]. The following substrates were used: Ca(OH)_2_ (≥99.5 wt%, POCH, Gliwice, Poland), H_3_PO_4_ (85.0 wt%, POCH, Gliwice, Poland), and medium-molecular-weight chitosan (~100,000 kDa, DD ≥ 75.0 wt%, Sigma-Aldrich, St. Louis, MO, USA). HAp-CTS hybrids in the form of powder and granules (300–400 μm) were applied as an aggregate in developed materials.

Five types of biomicroconcretes were prepared by mixing the solid phase, i.e., α-TCP, HAp-CTS materials, and polymeric additives with the liquid phase. As polymeric additives, sodium alginate (SA, POCH, Gliwice, Poland) or hydroxypropyl methylcellulose (HPMC, Alfa Aesar, Tewksbury, MA, USA) was used. The polymeric modifiers SA and HPMC were introduced in the form of powder directly during the cement paste mixing. As a liquid phase, a 5 wt% pectin gel in distilled water was used. A low esterified amidated pectin from citrus peels (CUL) was kindly delivered by Herbstreith & Fox (Herbstreith & Fox, Werder (Havel), Germany). The initial composition of the prepared biomicroconcretes, as well as a liquid to powder ratio (L/P), are presented in Table 1.

### 2.2. Methods

#### 2.2.1. Injectability and Setting Times

The injectability of the obtained materials was checked visually by injecting the cement paste through a 20 mL plastic syringe with a 2 mm nozzle (B. Brown, Melsungen, Germany) directly into the SBF solution preheated to 37 °C. The setting times (initial and final) of obtained biomicroconcretes were measured according to ASTM-C200-08 standard using a Gillmore apparatus (Humboldt, Norridge, IL, USA [34]. The results are presented as the average value of three measurements ± standard deviation (SD).

#### 2.2.2. Structural Studies

The crystalline phases were analysed by a powder X-ray diffractometer (XRD, D2 Phaser, Bruker, Ballerica, MA, USA). An XRD analysis was performed using CuK-α radiation (1.54 Å) at 30 kV and 10 mA. The intensity was recorded in a 2θ range from 10° to 90° at 0.04° intervals with a scanning speed of 2.5° min^−1^. The crystalline phases were identified by comparing the experimental diffractograms to the Joint Committee on Powder Diffraction Standards: α-TCP (JCPDS 00-009-0348) and hydroxyapatite (HAp; JCPDS 01-076-0694). A quantitative phase composition analysis based on Rietveld refinement was performed using Profex software (Version 4.3.5., Nicola Döbelin, Solothurn, Switzerland). The identification and quantification of the crystalline structures of materials nonincubated and incubated in simulated body fluid (SBF) at 37 °C were made after 7 days of setting and hardening.

Structural analyses were performed by Fourier transform infraRed spectroscopy (FTIR), within the scanning range 400–4000 cm^−1^ and resolution of 4 cm^−1^ using a BioRad FTS 6000 spectrometer (Vertex 70&70v, Bruker, Ballerica, MA, USA). The band positions of each result were measured according to the center of weight. The baseline correction, normalisation, and spectra analyses were performed using the Spectragryph software (Vwrsion v1.2.15, Friedrich Menges, Oberstdorf, Germany).

#### 2.2.3. Microstructure

The microstructure observations of the fractured samples were performed with the use of scanning electron microscopy (SEM, PhenomPure, Thermo Fisher Scientific, Waltham, MA, USA). In order to evaluate bioactive potential, the materials’ surface after 7 days of incubation in SBF at 37 °C was also assessed. Before the examination, the samples were coated with a thin gold film using a low deposition rate.

#### 2.2.4. Mechanical Strength

The compressive strength was examined using the universal testing machine (Instron 3345, Instron, Norwood, MA, USA). The cylindrical biomicroconcrete samples (6 mm × 12 mm) were subjected to uniaxial compression with a crosshead speed of 1.0 mm min^−1^. The biomicroconcrete samples were prepared and stored in air at 37 °C for 1 week. For comparison, samples after 7 days of incubation in SBF at 37 °C were also tested. The results of the compressive strength were presented as the average value of minimal ten measurements ± standard deviation (SD).

#### 2.2.5. Chemical Stability and Bioactivity In Vitro

In order to evaluate the chemical stability and bioactivity of the obtained materials, the cylindrical samples (12 mm × 6 mm) were placed in sterile containers filled with 40 mL of SBF or distilled water and stored at 37 °C for 28 days. The SBF solution was prepared according to Kokubo’s protocol [1,35]. The chemical stability of the obtained samples was assessed by measuring the pH of SBF and the ionic conductivity of the distilled water around incubated samples in the function of time using a pH/conductometer (H198129 Combo, Hanna, Smithfield, RI, USA). Measurements were performed at 1, 3, 7, and 28 days of incubation. Each measurement was performed triple times for each material. Results were expressed as the average value of three measurements ± standard deviation (SD).

#### 2.2.6. Statistics

A statistical analysis was performed using one-way ANOVA with a posthoc Tukey HSD (Honestly Significant Difference) test for comparing multiple treatments (* means the statistically significant difference between the results, *p* > 0.05). An analysis was performed with OriginPro 2021 software (Version 2021, OriginLab Corporation, Northampton, MA, USA).

## 3. Results and Discussion

### 3.1. Injectability and Setting Times

**Figure 1:**
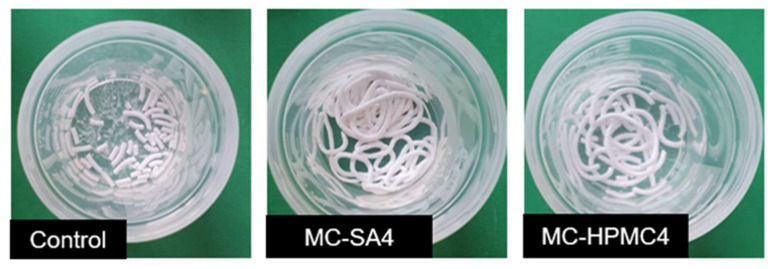
Obtained materials: Control, MC-SA4 and MC-HPMC4 immediately after extrusion from the syringe to SBF.

The novel biomicroconcrete-type biomaterials possessed excellent injectability. No phase separation was observed during the cement paste extrusion from the syringe to SBF. Moreover, it was observed that all developed materials were characterized by high cohesion and maintained their initial shape after extrusion, indicating washout resistance. However, the material MC-SA4, where the polysaccharide additive of 4 wt% of SA was applied, possessed the best injectability and cohesion in comparison to the others (Figure 1). No significant differences among the material groups containing the sodium alginate additive (MC-SA2, MC-SA4) and hydroxypropyl methylcellulose (MC-HPMC2, MC-HPMC4) were observed.

Outstanding surgical handiness was achieved through the use of natural polymers in the cement compositions. The polymeric additives increased the viscosity of the cement pastes and improved their injectability and cohesion. The ability of SA and HPMC to form hydrogels resulted in increased water resistance during the incubation in SBF, and the pectin crosslinking led to better integrity of the injected paste. Similar observations were reported in previous studies where polymeric additives were applied [18,28,36].

The setting times of the obtained cement-type materials depended on the polymeric additives and varied between 28 and 37 min (initial setting time) and more than 60 min (final setting time) (Table 2).

The results show that the addition of polysaccharides increased the initial cement setting time. Furthermore, the initial setting times of materials containing 2 wt% (MC-SA2 and MC-HPMC2) and 4 wt% of the polymeric additive (MC-SA4 and MC-HPMC4) were similar in the subgroups. The setting time determined the time in which material was completely hardened. The process that enables setting and hardening is α-TCP hydrolysis. α-TCP contained in the biomicroconcretes reacts with water and forms the calcium-deficient apatite, according to the following Equation (1) [37,38]:

3Ca_3_(PO_4_)_2_ + H_2_O → Ca_9_(PO_4_)5(HPO_4_)OH, (1)

Several factors influence the setting reaction of biomicroconcretes. The most important are: the amount of the highly reactive α-TCP phase in material, the presence of the setting accelerator in the liquid phase, the kind of polymeric additive in the liquid phase, and, finally, the presence of polymeric additives in the powder phase of the material [39]. The implantation of CPCs is a complex process with a brief window. Clinically, quick setting times can make the cement paste difficult to apply. Alternately, for the cement formulations with long setting times, the possibility of material washout constitutes a problem. The initial and final setting times of the obtained biomicroconcretes were higher than recommended in the literature (4–8 min for initial, up to 15 min for final) [40]. However, there are some commercially used bone substitutes characterized by longer than recommended setting times (i.e., Bone Source^®^: t_I_ = 10–15 min, t_F_~4 h; Norian SRS/CRS^®^: t_I_ = 10 min, t_F_~12 h) [41,42]. The presence of powder-added polymeric additives both in the form of sodium alginate (MC-SA2, MC-SA40) and hydroxypropyl methylcellulose (MC-HPMC2, MC-HPMC4) results in the elongation of the setting times of the obtained materials but also improves their cohesion. Furthermore, the amount of polymeric additive has an impact on the setting process. The higher the number of polysaccharides used, the longer the setting time was. These results are consistent with studies of Czechowska et al. [19]. In order to shorten the setting times, it would be essential to use a setting accelerator (i.e., disodium hydrogen phosphate Na_2_HPO_4_) [19,43]. Although the setting times of the obtained materials were longer than recommended, the presence of chitosan, citrus pectin, and other polymeric additives ensured good cohesion of the final materials, preventing them from disintegrating on contact with SBF. Moreover, the presence of the above-mentioned polysaccharides interactions and the setting of α-TCP powder caused the initiation of the dual setting process in the obtained biomicroconcretes. This process is based on the hydrolysis of α-TCP and polymer crosslinking simultaneously [44]. Chitosan and pectin interactions were studied previously by Marudova et al. [24], and they are based on the polyelectrolyte nature of those polymers. Chitosan as a natural polycation interacts with polyanionic pectin [27,45]. In addition, an idea for using other polysaccharides such as SA and HPMC as powder additives to biomicroconcretes was also motivated by the occurrence of interactions between them. Polyanionic sodium alginate may interact with polycationic chitosan and in the presence of calcium ions additionally crosslink, which will lead to a rigid ionic gel formation [14]. It also helps to form homogenous materials with beneficial mechanical properties [9,10].

### 3.2. Structural Studies

The materials phase composition was determined by XRD studies, whereas FTIR allowed the examination of the biomicroconcrete’s chemical composition. The initial α-TCP powder composed of α-TCP phase (98.0 wt%) and a small amount of hydroxyapatite (2.0 wt%). The HAp-CTS hybrid materials were composed of hydroxyapatite as the only crystalline phase. In the case of biomicroconcretes, diffractograms revealed the presence of two different crystalline phases corresponding to α-TCP and HAp, and their proportion varied according to the environment in which the samples were kept. The detailed phase composition is presented in Table 3.

**Figure 2:**
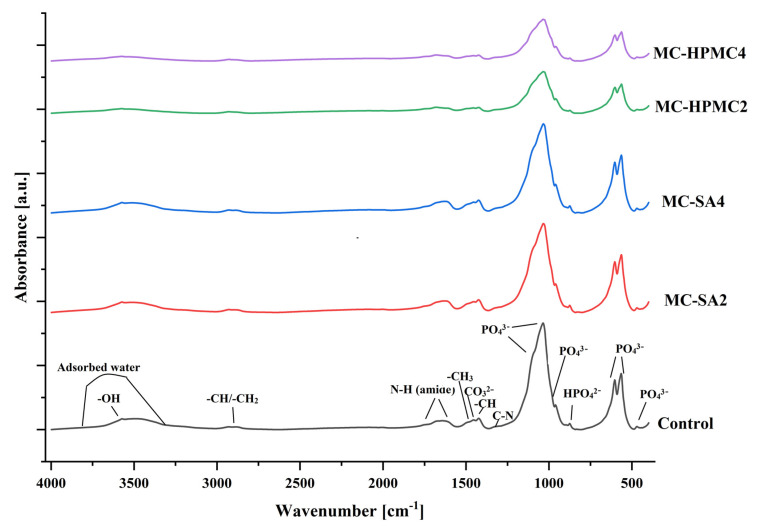
FT-IR spectra of obtained biomicroconcretes: Control, MC-SA2, MC-SA4, MC-HPMC2, and MC-HPMC4.

The diffractograms of biomicroconcretes revealed two crystalline phases, i.e., α-TCP and hydroxyapatite. In addition, the amorphous halos originating from polymers were present. Very similar results were obtained in previous studies on hybrid HAp-CTS materials [28,33]. Moreover, the analysis of the phase composition after material incubation in SBF confirmed that α-TCP almost completely hydrolyzed to hydroxyapatite (Table 3). As in a simulated environment, the α-TCP phase is thermodynamically metastable and spontaneously hydrolyzes to nonstoichiometric hydroxyapatite [40]. It is suggested that slight differences observed in the phase composition of individual materials were caused by the presence of polymer additives in the biomicroconcretes. The outcome of the FTIR measurements supports the results of the XRD studies. The FTIR analysis of the studied materials confirmed the presence of functional group characteristic for calcium phosphates and polysaccharides, such as chitosan or pectin (Figure 2). Infrared spectra of biomicroconcretes after setting and hardening revealed the presence of characteristic bands at 470, 563, 602, 958 as well as 1101 cm^−1^ that were assigned to the PO_4_^3−^ groups. The CO_3_^2−^ group forms a weak peak between 870 and 880 cm^−1^ (note: it overlaps here with HPO_4_^2−^) and a more intensive peak at 1424 cm^−1^ (note: overlapping with -CH bending). The absorption band with a maximum at 2928 cm^−1^ can be attributed to alkyl C-H stretching. The band at around 1649 cm^−1^ confirms the presence of N-H bending modes of the primary amine. Furthermore, the band around 1315 cm^−1^ corresponds to C-N stretching. Spectra also revealed a band at 3573 cm^−1^ which is attributed to O-H stretching vibrations, and a wide band around 3500 cm^−1^ indicates the presence of residual water in the studied samples. A low concentration of polymeric additives (i.e., sodium alginate and hydroxypropyl methylcellulose) in the cements, as well as an overlapping of bands, may explain the lack of visible additional bands assigned to these polysaccharides (i.e., C = O). Stretches typical for carboxylic groups at 1610 cm^−1^ (SA) [46] or esters around 3368, 1737, 1227, and 1377 cm^−1^ (HPMC) [47] were not visible.

### 3.3. Microstructure

**Figure 3:**
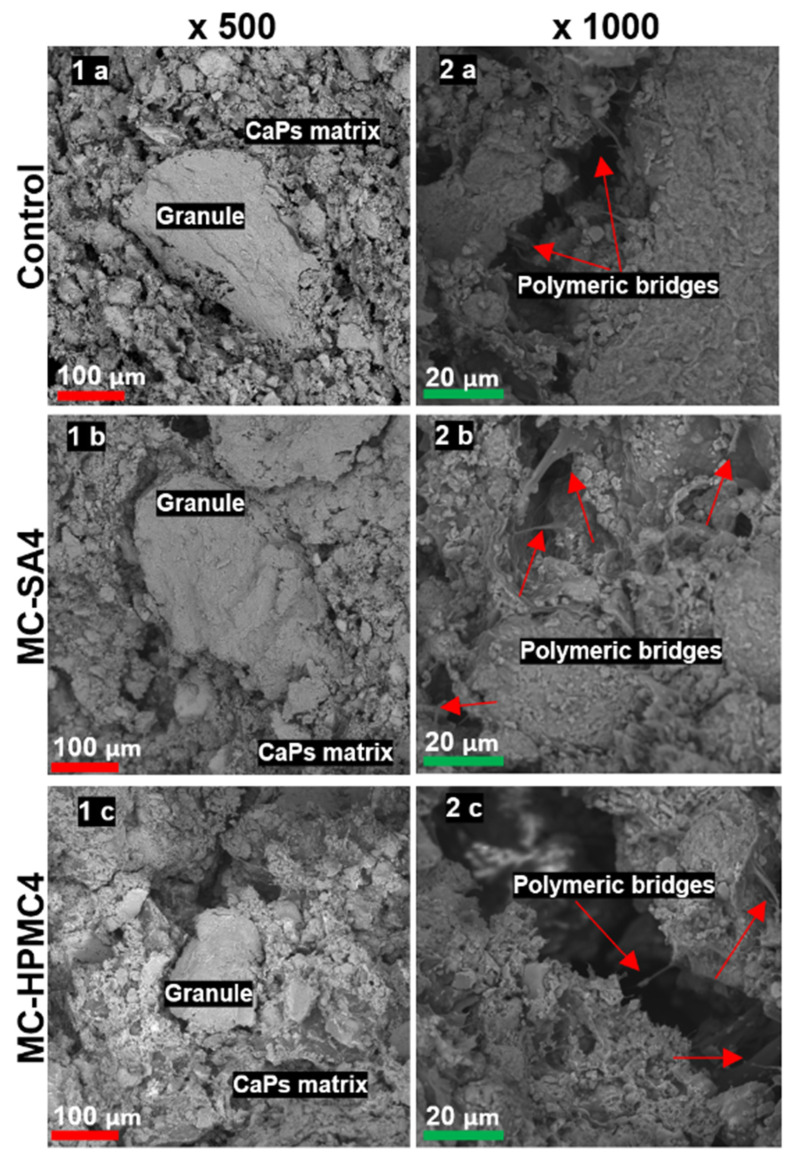
SEM microphotographs of cross-sections of obtained materials. Magnifications 500× (**1a**–**1c**) and 1000× (**2a**–**2c**).

SEM observations of the obtained composites after seven days of drying in air revealed that the materials possessed compact, homogenous microstructure formed by hybrid hydroxyapatite-chitosan granules and a cementitious matrix with visible micropores. The microstructure of hybrid HAp-CTS granules and biomicroconcretes was composed of calcium phosphates (CaPs) agglomerated matrix (Figure 3(1a–1c)) and polymers. The polymers present in developed materials were observed in the form of a polymeric film adhering to the calcium phosphate grains as well as polymeric bridges between the granules (Figure 3(2a–2c)). Characteristic polymeric bridges between granules and pectin present in the matrix were observed by others. For example, Zima et al. [33] developed hybrid HAp/CTS granules, while Mickiewicz et al. [48] added various water-soluble polymers to commercial calcium phosphate bone substitutes. The presence of numerous characteristic chitosan bridges is evidence of the interactions among hybrid HAp/CTS materials, citrus pectin, and probably polymeric additives [13,24]. Similar microstructures were observed previously by Czechowska et al. [32] where the addition of chitosan to the liquid phase of α-TCP-based bone cements was investigated. 

**Figure 4:**
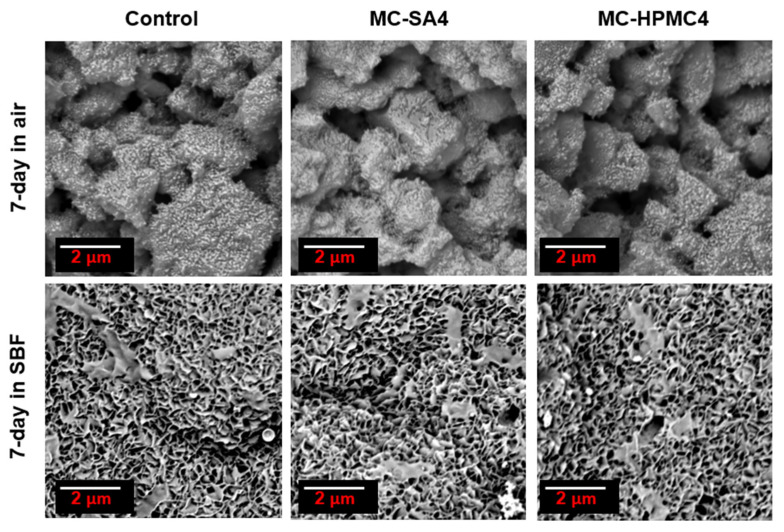
SEM microphotographs of biomicroconcretes surface after 7-day incubation in air or SBF.

After seven days of incubation in SBF, the surfaces of biomicroconcretes were completely covered by apatitic structures (Figure 4). The materials were characterized by similar microstructures regardless of the content of the polymeric additive. Hybrid granules were in intimate contact with the cementitious matrix, therefore all obtained composites are characterized by good mechanical properties and cohesion in the presence of a simulated biological environment.

### 3.4. Mechanical Strength

**Figure 5:**
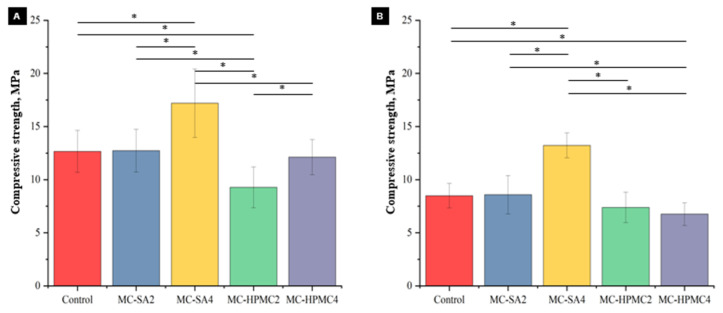
Compressive strength of biomicroconcretes after 7 days of drying in air (**A**) and after 7 days of incubation in SBF (**B**) (*—statistically significant difference, *p* < 0.05).

The results of compressive strength tests, both after seven days of drying in air (Figure 5A) and after seven days of incubation in SBF at 37 °C (Figure 5B) are shown bellow.

The compressive strength of the obtained cements ranged from 9.3 ± 2.1 to 17.2 ± 2.6 MPa for materials after seven days of setting and hardening and from 6.6 ± 1.2 to 13.2 ± 1.2 MPa for materials after incubation in SBF. A high compressive strength of the biomicroconcretes was obtained due to the presence of a dual setting system (α-TCP hydrolysis and interaction between sodium alginate and other polymeric additives in the material matrix composition) [24]. The mechanical properties of the obtained biomicroconcretes result not only from their hybrid nature but also from the characteristics of the additional polymer. Sodium alginate, compared to HPMC in an aqueous solution, is characterized by greater rigidity and viscosity, which may have a direct impact on the final properties of the developed biomaterials [49,50]. The highest compressive strength value was recorded for the material MC-SA4, where the 4 wt% addition of sodium alginate was applied (up to 17.2 ± 3.4 MPa). The increased strength with adding sodium alginate compared to the control material was due to the presence of additional interactions between Ca^2+^ cations and anionic sodium alginate. Alternately, materials containing another polymeric additive, hydroxypropyl methylcellulose, were characterized by lower compressive strength. A similar negative influence of HPMC on the material strength was also reported by Perez et al. [51]. It is suggested that the weakening of the strength of the materials with the HPMC addition is caused by the fact that HPMC is a highly hygroscopic polymer [52], thus absorbed water is necessary for α-TCP hydrolysis. Nevertheless, compressive strength values similar to that of human cancellous bone (between 4 and 12 MPa) [53] were obtained. Thus, it makes the obtained biomicroconcretes suitable for low-load bearing applications.

### 3.5. Chemical Stability and Bioactivity In Vitro

**Figure 6:**
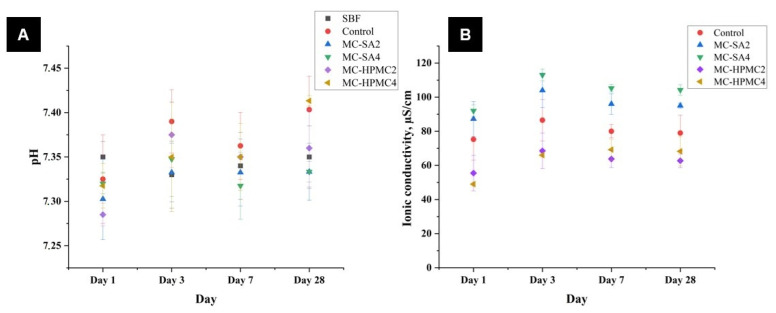
pH vs. time of incubation in SBF (**A**) and ionic conductivity vs. time of incubation in distilled water (**B**).

Chemical stability studies are one of the important tests for considering implantable materials for medical applications. Incubating the sample in SBF is considered as a first step for evaluating material characteristics and understanding their degradation mechanisms [54]. In order to evaluate chemical stability and bioactivity in vitro of the obtained biomicroconcretes, materials samples were incubated in simulated body fluid. Figure 6A shows the pH changes of SBF during the immersion of the samples. The pH changes of SBF remained close to the physiological values and ranged from 7.22 to 7.41. The addition of polysaccharides in the form of sodium alginate (MC-SA2 and MC-SA4) or hydroxypropyl methylcellulose (MC-HPMC2and MC-HPMC4) only slightly influenced the solution’s pH values. Similar pH values of incubated CPCs were observed elsewhere [32,55]. Ionic conductivity during the incubation in distilled water of the control material was in the range of ~75–87 μS/cm. An additive in the form of sodium alginate (materials MC-SA2 and MC-SA4) caused an increase in ionic conductivity (~87–113 μS/cm), whereas for HPMC (materials MC-HPMC2 and MC-HPMC4), a decrease of ionic conductivity to ~49–69 μS/cm was observed (Figure 6B). This phenomenon can be explained by a higher degradation rate of SA than HPMC in aqueous solutions [18,56,57]. Ionic conductivity of all the obtained materials reached a plateau after seven days of incubation in distilled water.

The results show that polymeric additives did not affect the chemical stability of the developed biomicroconcretes incubated in SBF.

The bioactive potential of materials was estimated through the incubation in SBF according to Kokubo’s protocol [35]. The SEM observations show that the evenly distributed, bone-like apatite layer was present on the sample’s surface after seven days of incubation in SBF at 37 °C (Figure 4). The materials MC-SA2 and MC-HPMC2 possessed similar microstructure. The study revealed the presence of needle-like crystal structures forming an apatite-like layer after the seven-day incubation in SBF. The presence of apatite forms indirectly indicates the high bioactive potential of the obtained materials. The cement-type materials with hybrid HAp/CTS granules have been proven to be biocompatible in vivo by Zima et al. [18]. The polymeric additives used in the study had no adverse effects on the cells due to their beneficial biological properties. Thus, it may be assumed that the obtained biomaterials also will be biocompatible. To confirm this hypothesis further studies are necessary.

## 4. Conclusions

In this study, innovative biomicroconcretes based on highly reactive α-TCP powder, hybrid hydroxyapatite-chitosan hybrids (granules and powder), and citrus pectin gel were developed and examined. Moreover, the influence of polymeric additives in the form of sodium alginate and hydroxypropyl methylcellulose on the obtained materials was investigated. Newly developed biomicroconcretes differ significantly from materials previously described in the literature. Thus, in this paper, the main focus was on investigating the effect of polymer additives on the physicochemical properties of biomicroconcrete-type materials. The idea of using hybrid HAp-CTS granules as aggregates in developed biomaterials with the combination of the use of biopolymers resulted in novel composites characterized by beneficial properties of bioceramics (as calcium phosphates) and biopolymeric materials. It has been demonstrated that the addition of both natural polymers influenced the physicochemical properties of the composites. All obtained biomicroconcretes were characterized by excellent injectability and cohesion in simulated body fluid. It was also observed that the liquid phase in the form of gelled citrus pectin as well as the addition of the polymer influenced the material’s setting process. Moreover, it was observed that a polymeric additive in the form of 4 wt% of sodium alginate unlike hydroxypropyl methylcellulose caused the biomicroconcretes to strengthen (up to 17.2 MPa for nonincubated materials and 13.2 MPa for materials incubated in SBF). Unique mechanical properties can be connected to the dual setting system occurring in developed materials, where α-TCP hydrolysis and polymer crosslinking take place simultaneously. The formation of hydrogel complexes during the preparation of the cement pastes allowed for the interaction between the polymers present in the tested materials (i.e., interaction between polycationic and polyanionic polymers). In vitro studies revealed that developed materials were chemically stable and demonstrated bioactive potential by the formation of apatitic layers on their surfaces as early as seven 7 days after incubation in SBF. Thus, the obtained materials can be considered as potentially bioactive. The ionic conductivity changed depending on the presence of the polymeric additive used. Sodium alginate caused an increase in ionic conductivity, unlike hydroxypropyl methylcellulose, which is indirectly connected to the degradation process of these polymers. All these findings confirm the most beneficial influence of the addition of 4 wt% sodium alginate on the character of the obtained biomicroconcretes and pave the way to further in vitro and in vivo studies.

## Figures and Tables

**Table 1:** Initial compositions of developed materials.

Material Label	Solid (Powder) Phase (P)	Liquid Phase (L)	L/P (mL/g)
Control	25 wt% α-TCP: 35 wt% HAp/CTS granules: 40 wt% HAp/CTS powder	5 wt% CUL in distilled water (gel)	0.8
MC-SA2	Control + 2 wt% SA powder		
MC-SA4	Control + 4 wt% SA powder		
MC-HPMC2	Control + 2 wt% HPMC powder		
MC-HPMC4	Control + 4 wt% HPMC powder		

**Table 2:** The setting times of the biomicroconcretes.

Material	Initial, t_i_ [Min]	Final, t_f_ [Min]
Control	28 ± 2	>60
MC-SA2	33 ± 2	
MC-SA4	36 ± 3	
MC-HPMC2	34 ± 1	
MC-HPMC4	37 ± 2	

**Table 3:** Phase composition of tested biomicroconcretes.

Material Label	Phase Composition [wt%]			
	7 Days after Setting and Hardening		After 7 Days of Incubation in SBF (37 °C)	
	α-TCP	HAp	α-TCP	HAp
Control	25.0	75.0	2.0	98.0
MC-SA2	26.0	74.0	2.0	98.0
MC-SA4	29.0	71.0	3.0	97.0
MC-HPMC2	26.0	74.0	2.0	98.0
MC-HPMC4	28.0	72.0	1.0	99.0

## Data Availability

All the data are available within the manuscript.
